# Spermatogenesis and Morphology of the Male Reproductive Tract of the Turtle *Kinosternon scorpioides*

**DOI:** 10.1111/ahe.70101

**Published:** 2026-03-16

**Authors:** Vinicius Maia Ribeiro de Godoy, Alana Lisleia de Sousa, Mariapaz Dueñas Flórez, Antônio Francisco da Silva Lisboa Neto, Antonio Chaves de Assis Neto

**Affiliations:** ^1^ Department of Surgery Graduate Program in the Anatomy of Domestic and Wild Animals, School of Veterinary Medicine and Animal Science, University of São Paulo São Paulo Brazil; ^2^ Department of Clinic State University of Maranhão São Luís Brazil; ^3^ Federal University of Piauí (UFPI) Piauí Brazil

**Keywords:** anatomy, conservation, reproductive period, spermatogenesis, spermatozoa, ultrastructure

## Abstract

The scorpion mud turtle ( *Kinosternon scorpioides* ), a freshwater chelonian widely distributed in South and Central America, presents gaps in the microscopic and ultrastructural characterisation of its male reproductive system. This study provides a comprehensive characterisation of the testes, epididymides, and deferent ducts of adult males collected during the rainy reproductive season in the Brazilian Amazon. Morphometric parameters were recorded and showed a positive association between body weight and reproductive tract mass, supporting increased gonadal investment during the breeding period. Tissues were processed for light microscopy (LM), transmission electron microscopy (TEM), and scanning electron microscopy (SEM). Histological and ultrastructural analyses revealed active spermatogenesis, abundant germ cells in multiple developmental stages, and regionally specialised epithelia along the epididymis and deferent ducts. Spermatozoa exhibited preserved acrosomal, nuclear, mitochondrial and axonemal structures, indicating functional integrity and short‐term storage capacity within the male reproductive tract. These findings expand current knowledge of chelonian reproductive biology and provide essential baseline data to support assisted reproductive technologies and conservation strategies for *K. scorpioides* and related freshwater turtle species.

## 1 Introduction

The scorpion mud turtle ( *Kinosternon scorpioides* [Linnaeus, 1766]) is widely distributed throughout Central and South America (Berry and Iverson 2011; Vanzolini et al. 1980) and displays pronounced sexual dimorphism marked by differences in plastron morphology, tail length and head coloration (Avendaño et al. 2002; Berry and Iverson 2011; Silva Da Silva, dos Santos Braga, et al. 2021). Reproductive function in this species, as in many reptiles, is strongly influenced by environmental seasonality, particularly rainfall, humidity, and temperature, which regulate both gametogenesis and mating behaviour (Berry and Iverson 2011; Carvalho Viana, Almeida Rui, et al. 2014; Fernandes Araujo Chaves et al. 2020; Medeiros et al. 2024).

Despite its ecological and conservation relevance, available studies have focused primarily on gross morphology, with limited information on the histological and ultrastructural organisation of the male reproductive organs (Cardoso Carvalho et al. 2010; Sousa et al. 2014; Viana et al. 2014). This gap restricts deeper understanding of reproductive physiology, especially regarding seasonal activation and potential mechanisms of sperm storage.

Growing anthropogenic pressures, including habitat degradation, illegal harvesting and climate instability, underscore the need for comprehensive reproductive data. Such information can inform conservation strategies and guide the development of assisted reproductive technologies (ARTs), such as semen collection, cryopreservation and artificial insemination (Berriozabal‐Islas et al. 2020; Rodrigues et al. 2017).

Therefore, this study aimed to provide an integrated macroscopic, histological and ultrastructural characterisation of the testes, epididymides, and deferent ducts of male *K. scorpioides* during the rainy season, which corresponds to the reproductive period. These findings establish a foundation for advancing reproductive biology research and informing conservation strategies for this and related chelonian species.

## 2 Materials and Methods

### 2.1 Animals and Sample Collection

Ten adult male *scorpion mud turtles* were collected in the municipalities of São Bento (2.69583° S, 44.82139° W) and Pinheiro (2.52083° S, 45.08278° W), both located in the Baixada Maranhense microregion, Maranhão State, Brazil. One additional specimen originated from captivity at the Universidade Estadual do Maranhão (UEMA) (2.58045° S, 44.20756° W). These regions are characterised by a humid tropical climate and extensive floodplain areas, environmental features that may directly influence the biological parameters evaluated in the present study.

Specimens were captured under ICMBio/MMA licence (permit no. 33021‐4) and procedures were approved by the Ethics Committee on Animal Use of the School of Veterinary Medicine and Animal Science, University of São Paulo. Collections took place during the rainy season (January–February), coinciding with the species' reproductive peak.

Animals were pre‐anaesthetised with intramuscular xylazine hydrochloride (2%, 40 mg/kg) and ketamine hydrochloride (1%, 60 mg/kg), followed by euthanasia via intravenous sodium thiopental (2.5%, 60 mg/kg) administered through the cervical venous sinus, as described by Schumacher (Schumacher 1996). Morphometric data (body and testicular weights, carapace/plastron dimensions) were collected using a precision digital scale (SF‐400) and a digital calliper (JOMARCA).

Voucher specimens examined in this study are located in the Laboratory of Veterinary Anatomy, Universidade Estadual do Maranhão (UEMA), Brazil. Processed and paraffin‐embedded tissue samples are housed in the Laboratory of Applied Morphological Studies in Veterinary Medicine, Universidade de São Paulo (USP), Brazil.

### 2.2 Macroscopic Analysis

Following euthanasia, the coelomic cavity was accessed by disarticulating the bony bridge between the plastron and carapace using a DC‐130B‐5 electric microdrill. The male reproductive organs were examined in situ, and their morphology, coloration, positioning, and relational anatomy were documented.

### 2.3 Fixation and Processing for Light Microscopy

Fragments of testes, epididymides, and deferent ducts were fixed in Bouin's solution for 24 h, dehydrated in graded ethanol (70%–100%), cleared in xylene, and embedded in paraffin. Sections (5 μm) were obtained using a Leica RM2155 microtome, then stained with haematoxylin and eosin using standard protocols (Santos et al. 2012). Slides were mounted and analysed using an Olympus BX61VS microscope.

### 2.4 Scanning Electron Microscopy (SEM)

Samples fixed in 4% paraformaldehyde were rinsed in phosphate buffer, post‐fixed in 1% osmium tetroxide, and dehydrated through graded ethanol (50%–100%). After critical point drying (LEICA EM CPD300), specimens were mounted on stubs with carbon tape and sputter‐coated with silver (EMITECH K550). Images were acquired using a LEO 435VP scanning electron microscope.

### 2.5 Transmission Electron Microscopy (TEM)

Tissues fixed in 2.5% glutaraldehyde were rinsed in PBS (0.1 M, pH 7.4), post‐fixed in 1% osmium tetroxide, and dehydrated in ascending ethanol concentrations. Samples were infiltrated with a 1:1 mix of propylene oxide and Spurr's resin (Electron Microscopy Sciences, USA), rotated for 12–16 h, then transferred to pure resin and polymerised at 69°C for 72 h. Semi‐thin sections (1 μm) were stained with toluidine blue; ultrathin sections (~60 nm) were stained with uranyl acetate and lead citrate before TEM imaging.

### 2.6 Statistical Analysis

Descriptive statistics were calculated for all morphometric and testicular variables and expressed as mean ± standard deviation (SD). The coefficient of variation (CV%) was calculated to assess relative variability among parameters. Differences between right and left testis weight were evaluated using the Wilcoxon signed‐rank test. Associations between body size (body weight and carapace length) and testicular weight were assessed using Spearman's rank correlation coefficient. Statistical significance was set at *p* < 0.05.

## 3 Results

### 3.1 Gross Anatomy of the Male Reproductive Tract

For each individual, the following morphometric variables were recorded: body weight, carapace length and width, plastron length and width, body height, and testis weight (left and right). Mean values and standard deviations are presented in Table 1.

**TABLE 1:** Morphometric and testicular measurements of adult male *K. scorpioides* (mean ± SD).

A	BW (g)	CL (cm)	CW (cm)	PL (cm)	PW (cm)	H (cm)	RTW (g)	LTW (g)
1	341.04	14.11	8.63	12.96	6.32	3.68	1.18	1.02
2	453.24	16.00	9.26	14.45	7.05	5.31	2.55	2.17
3	394.48	15.22	9.11	13.22	7.11	5.12	0.75	0.75
4	355.28	14.61	9.02	12.09	5.76	4.60	1.75	1.55
5	274.20	14.87	8.52	12.26	5.92	4.56	0.79	0.70
6	340.75	15.83	9.67	13.06	7.34	5.62	1.95	2.09
7	160.57	11.10	7.17	9.88	4.83	4.11	0.13	0.14
8	179.77	11.40	7.46	9.98	4.47	4.16	0.15	0.22
9	191.56	9.00	7.20	8.57	4.52	4.34	0.28	0.33
10	195.00	11.83	7.66	10.37	5.16	3.98	0.59	0.41
11	374.82	14.91	9.09	12.96	6.27	4.66	2.48	2.35
Mean ± SD	296.43 ± 100.80	13.53 ± 2.31	8.44 ± 0.90	11.80 ± 1.82	5.89 ± 1.04	4.56 ± 0.59	1.15 ± 0.90	1.07 ± 0.83
CV (%)	34.01	17.06	10.71	15.42	17.68	13.04	78.72	78.07

*Note:* Values are expressed as mean ± SD.

Abbreviations: A, animal; BW, body weight; CL, carapace length; CV, coefficient of variation (%); CW, carapace width; H, body height; LTW, left testis weight; PL, plastron length; PW, plastron width; RTW, right testis weight.

Adult male *Kinosternon scorpioides* examined in this study exhibited moderate variation in body size and testicular mass (Table 1). Body weight ranged from 160.57 to 453.24 g (296.43 ± 100.80 g), reflecting the inclusion of individuals at different stages of adult somatic development. Carapace length (CL) varied between 9.00 and 16.00 cm (13.53 ± 2.31 cm), while carapace width (CW) ranged from 7.17 to 9.67 cm (8.44 ± 0.90 cm), indicating relatively consistent shell proportions. Plastron length (PL), plastron width (PW), and body height (H) also showed limited variability (CV = 13%–18%), suggesting morphological homogeneity typical of adult males.

In contrast, testicular weights exhibited markedly higher coefficients of variation (~78%) than external morphometric parameters (Table 1). Right testis weight (RTW) ranged from 0.13 to 2.55 g (1.15 ± 0.90 g), while left testis weight (LTW) ranged from 0.14 to 2.35 g (1.07 ± 0.83 g). No significant difference was detected between right and left testis weights (Wilcoxon signed‐rank test, *p* = 0.114), indicating the absence of lateral asymmetry in testicular development.

**FIGURE 1:**
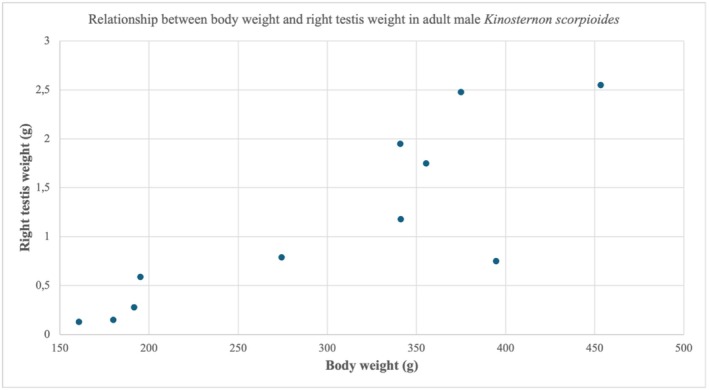
Relationship between body weight and right testis weight in adult male *Kinosternon scorpioides*. Scatter plot showing the positive association between body weight and right testis weight (Spearman's *ρ* = 0.84, *p* < 0.01).

Both right and left testis weights were strongly correlated with body weight and carapace length (Spearman's *ρ* = 0.83–0.86, *p* < 0.01), indicating increased gonadal investment in larger males. Figure 1 illustrates the positive relationship between body weight and right testis weight; similar significant correlations were detected for the left testis. This pattern is compatible with seasonal reproductive activation, in which gonadal size may fluctuate more markedly than external morphometry. These morphometric and gonadal parameters provide a baseline for adult male *K. scorpioides* during the rainy reproductive period and support subsequent histological and ultrastructural findings of intense spermatogenic activity.

**FIGURE 2:**
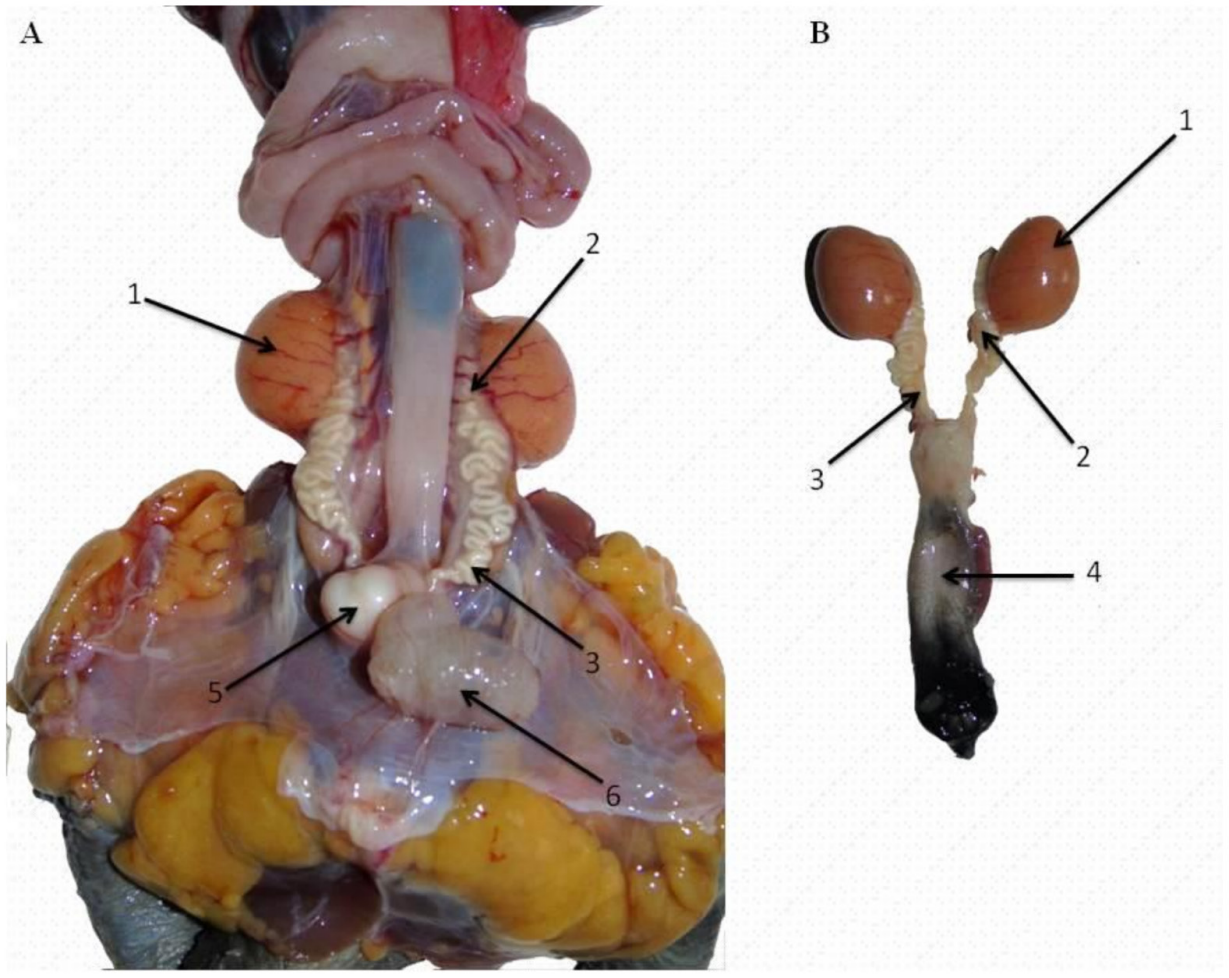
Gross anatomy of the male reproductive tract of *Kinosternon scorpioides* . Black arrows indicate: (1) testis, (2) epididymis, (3) deferent duct, (4) penis, (5) penile root, (6) urinary bladder. (A) Left: male reproductive tract in situ, showing its anatomical relationship with adjacent structures, including blood vessels, the gastrointestinal tract and adipose tissue. (B) Right: isolated male reproductive tract.

As shown in Figure 2, the male reproductive tract consisted of paired testes, epididymides, deferent ducts, and a single penis. Testes were globular, smooth‐surfaced, and yellow to orange in colour, with the right testis located slightly more cranially than the left. The epididymides, emerging from the cranial poles of the testes, were highly coiled and adhered medially without macroscopic differentiation into caput, corpus or cauda. Each epididymis connected to a deferent duct, which coursed dorsocaudally to empty into the cloaca. No ampullary expansion was observed. The deferent ducts were closely associated with the ureters and conveyed both spermatozoa and urine.

### 3.2 Transmission and Scanning Electron Microscopy

**FIGURE 3:**
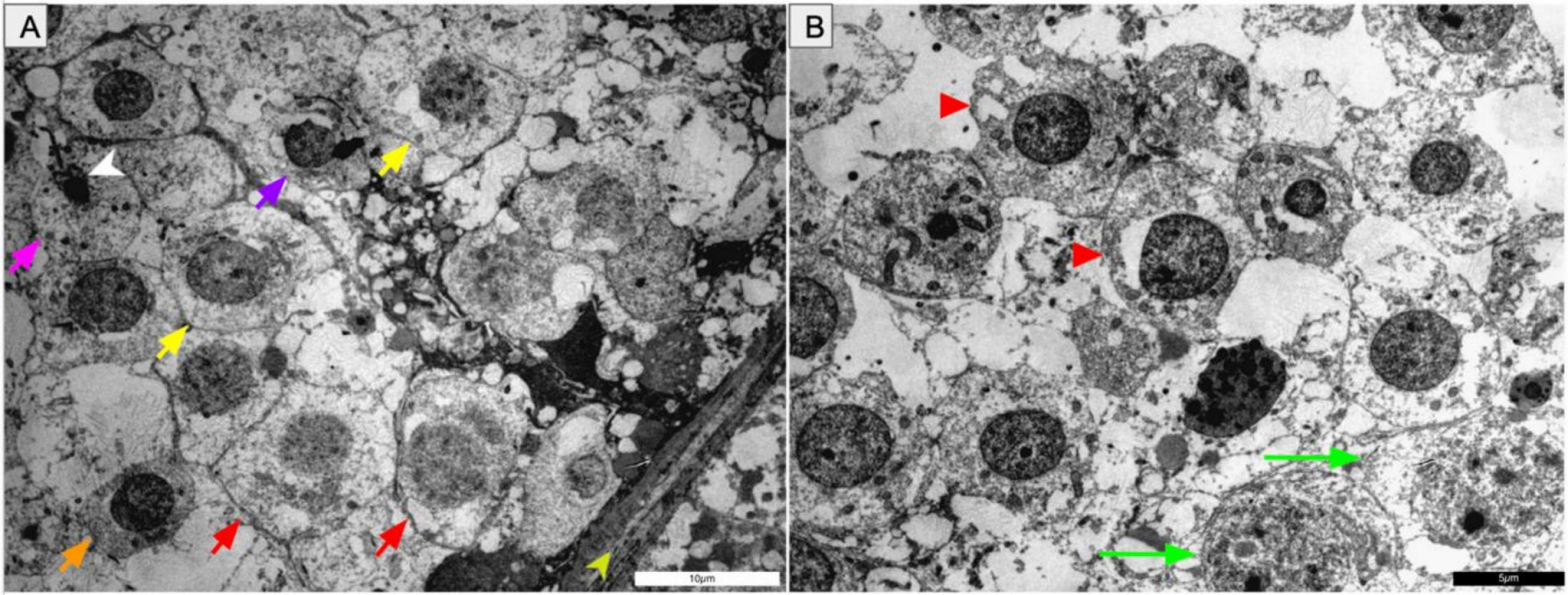
Transmission electron microscopy (TEM) of the testis of *K. scorpioides* . (A) Germ cells in different stages: light spermatogonium (red arrow), primary spermatocyte (orange arrow), secondary spermatocyte (yellow arrow), spermatid stage I (purple arrow), spermatid stage II (pink arrow), and sperm head (white arrowhead). Basal membrane (light green arrowhead). (B) Dark spermatogonium (red arrowhead) and Sertoli cell (green arrow). Scale bars: (A) 10 μm; (B) 5 μm.

Ultrastructural analysis of the testes revealed active spermatogenesis, evidenced by seminiferous epithelium containing spermatogonia (light and dark), primary and secondary spermatocytes, round spermatids, and spermatozoa within the lumina (Figure 3). Sertoli cells were identifiable along the basal lamina. These observations confirm a functionally active reproductive state during the rainy season.

**FIGURE 4:**
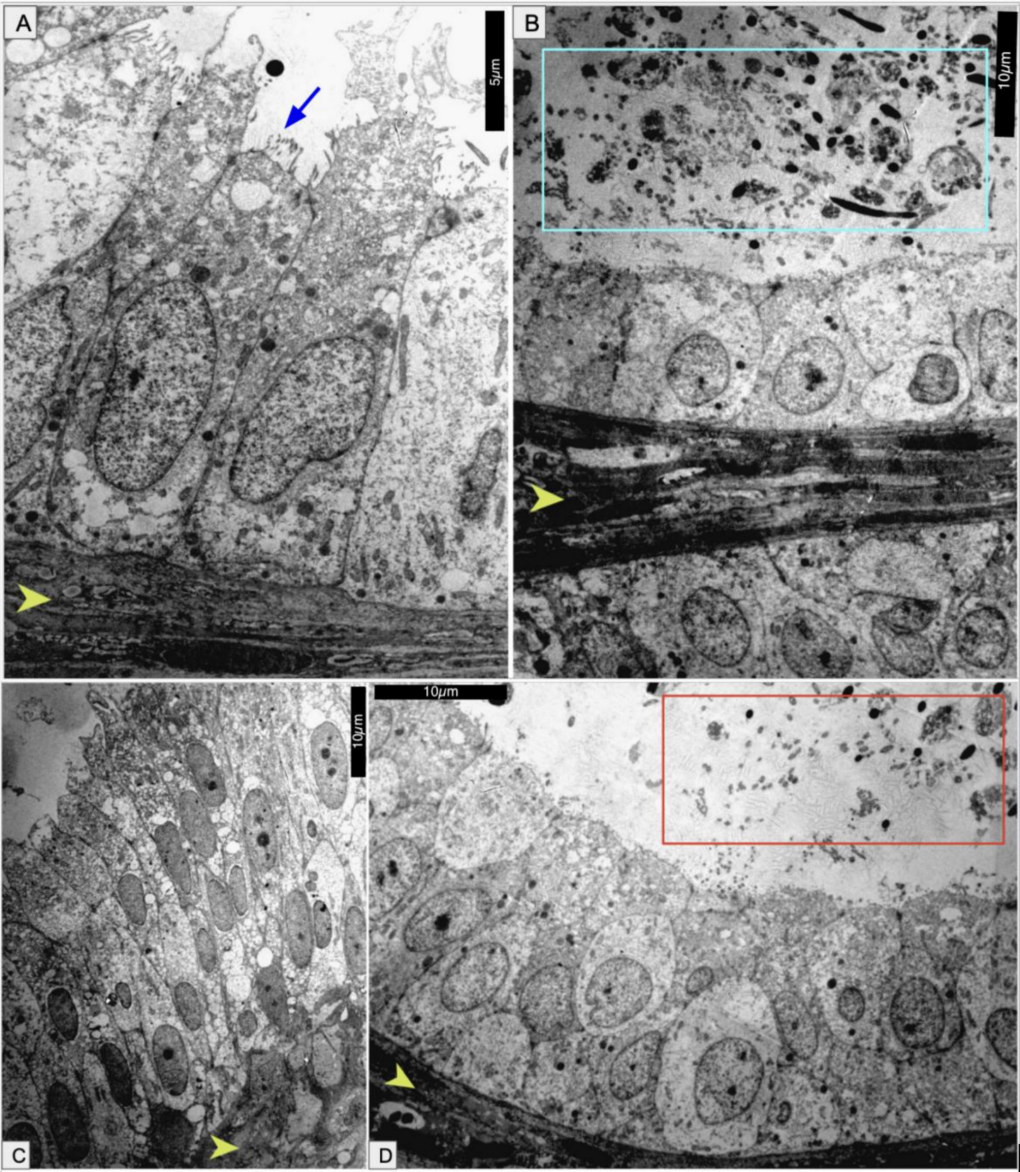
TEM images of epididymal epithelial variation in *K. scorpioides* . (A) Simple columnar epithelium with basal membrane (arrowhead) and stereocilia (blue arrow). (B) Simple cuboidal epithelium with basal membrane (arrowhead) and luminal spermatozoa (blue square). (C) Pseudostratified columnar epithelium with basal membrane (arrowhead). (D) Pseudostratified cuboidal epithelium with basal membrane (arrowhead) and luminal spermatozoa (red square). Scale bars: (A) 5 μm; (B–D) 10 μm.

The epididymides exhibited marked epithelial heterogeneity, including simple cuboidal, simple columnar, and pseudostratified epithelia, with or without stereocilia, lining ducts containing luminal spermatozoa (Figure 4). This diversity indicates regional specialisation within the epididymal segments associated with sperm maturation and transport.

**FIGURE 5:**
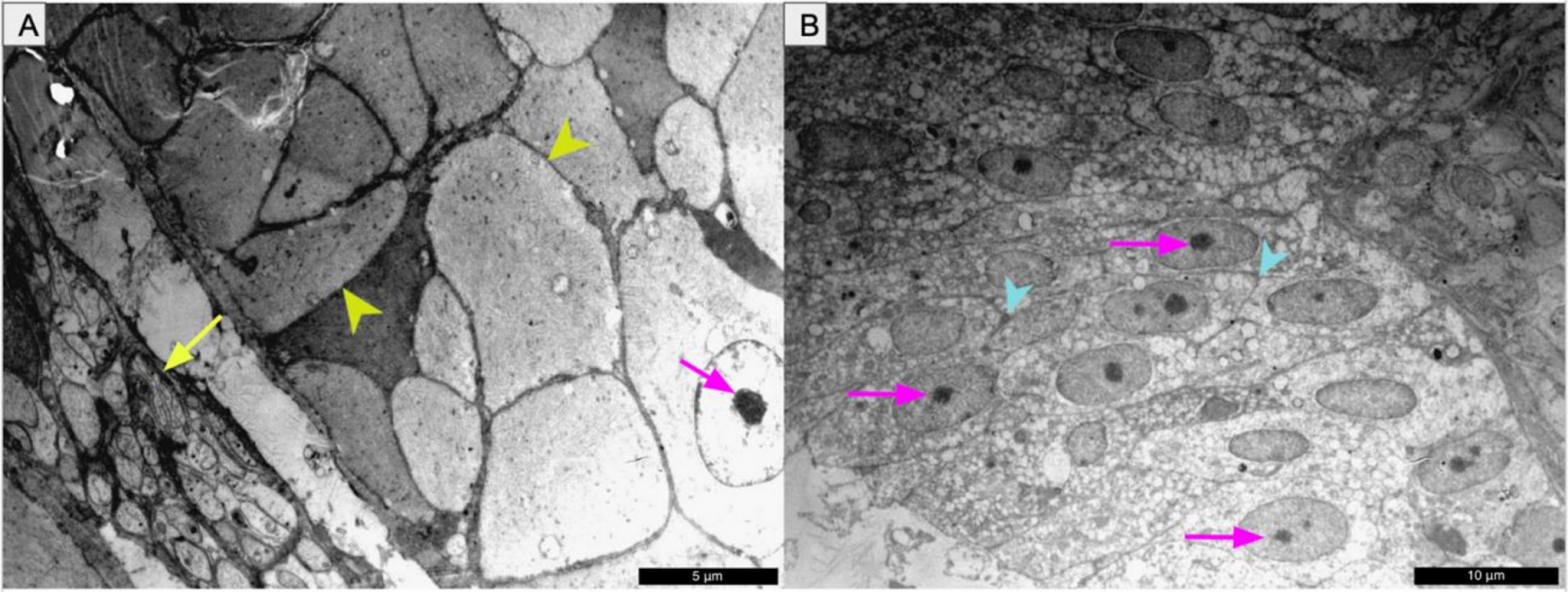
TEM images of the deferent duct epithelium in *K. scorpioides* . (A) Lipid vesicles (yellow arrowheads), rough endoplasmic reticulum (yellow arrow), and nucleoli (pink arrow). (B) Nucleus with prominent nucleoli (pink arrow) and plasma membrane (light blue arrowhead). Scale bars: (A) 5 μm; (B) 10 μm.

In the deferent ducts, epithelial cells displayed prominent nucleoli and cytoplasmic organelles such as rough endoplasmic reticulum and lipid inclusions (Figure 5), indicative of secretory and absorptive capacity. Spermatozoa were present within the lumina, and the walls exhibited a gradual increase in smooth muscle thickness, consistent with enhanced storage and propulsion functions.

**FIGURE 6:**
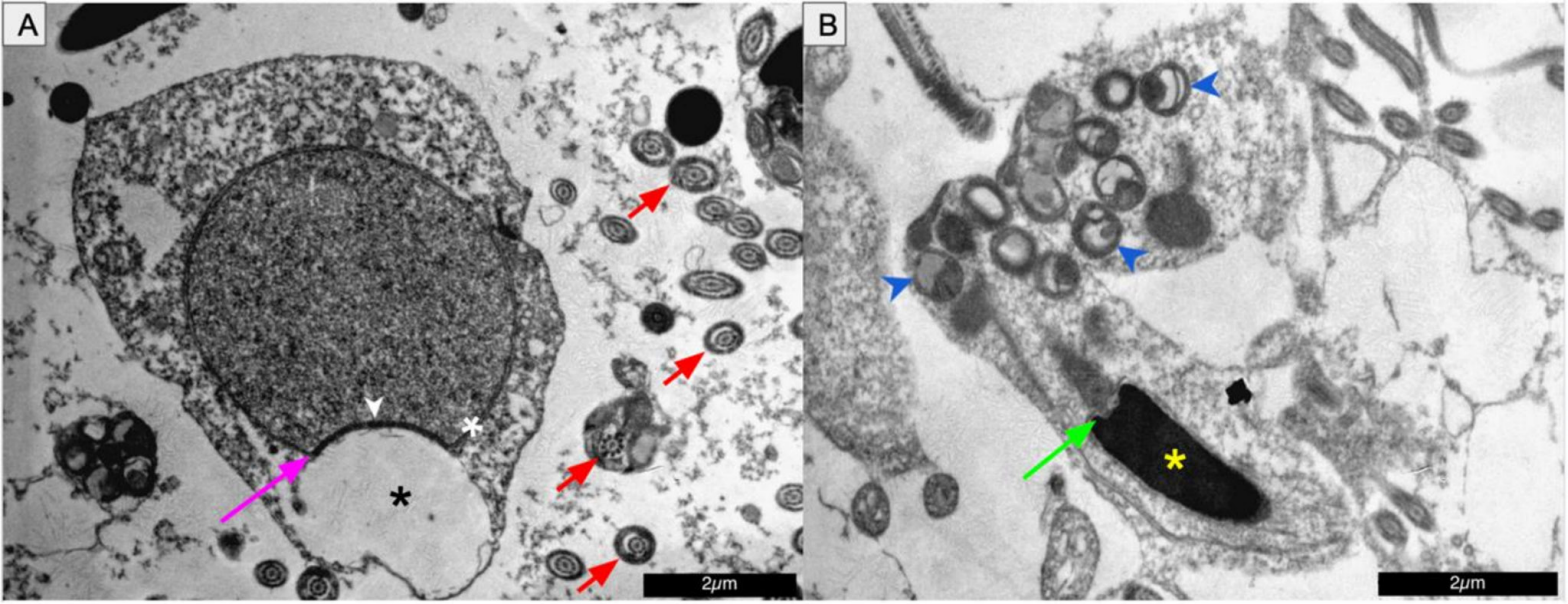
Ultrastructure of spermatids and spermatozoa in *K. scorpioides* (TEM). (A) Proacrosomal vesicle (black asterisk) adjacent to the nuclear membrane (white asterisk), subacrosomal granule (white arrowhead), and cross‐sections of flagella showing the ‘9 + 2’ microtubule pattern (red arrow). (B) Longitudinal view of a mature spermatozoon showing nuclear region (yellow asterisk), mitochondrial sheath (blue arrowheads), and caudal nuclear depression (green arrow). Scale bars: 2 μm.

Ultrastructurally, spermatozoa exhibited well‐defined acrosomal vesicles, centriolar complexes, mitochondrial sheaths, and flagella with the classical ‘9 + 2’ axonemal microtubule arrangement (Figure 6). These features are indicative of preserved motility and fertilisation competence.

**FIGURE 7:**
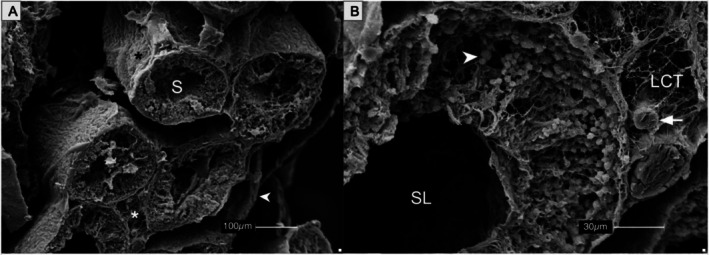
Scanning electron microscopy (SEM) of seminiferous tubules from *K. scorpioides* . (A) Seminiferous tubules (S) surrounded by the tunica albuginea (arrowhead) and interstitial connective tissue (asterisks). (B) Seminiferous lumen (SL) bordered by loose connective tissue (LCT), with arterioles (arrow) and developing germ cells (arrowheads). Scale bars: (A) 100 μm; (B) 30 μm.

**FIGURE 8:**
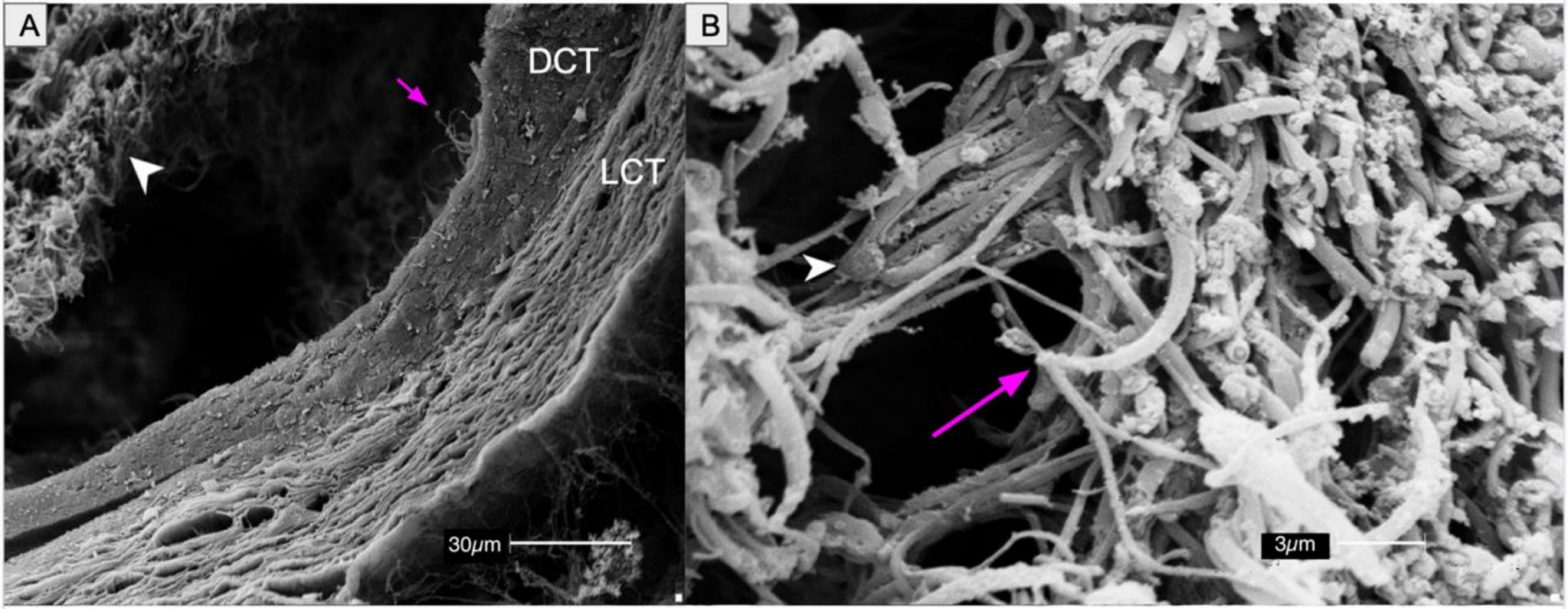
SEM images of the epididymis of *K. scorpioides* . (A) Epididymal duct surrounded by dense connective tissue (DCT) and loose connective tissue (LCT). Spermatozoa (arrowhead) and stereocilia (arrow). (B) Cluster of spermatozoa showing heads (arrowhead) and flagella (arrow). Scale bars: (A) 30 μm; (B) 3 μm.

Scanning electron microscopy (SEM) confirmed the cytoarchitecture of seminiferous tubules, the structural complexity of the epididymis, and the presence of sperm clusters within the ducts (Figures 7 and 8). Ducts were surrounded by dense and loose connective tissue, and stereocilia projected into sperm‐rich lumina.

The presence, distribution, and preservation of mature spermatozoa throughout the testes, epididymides, and deferent ducts support the occurrence of peak seasonal reproductive activity and indicate the capacity for sperm storage during this period.

### 3.3 Histological Analysis

**FIGURE 9:**
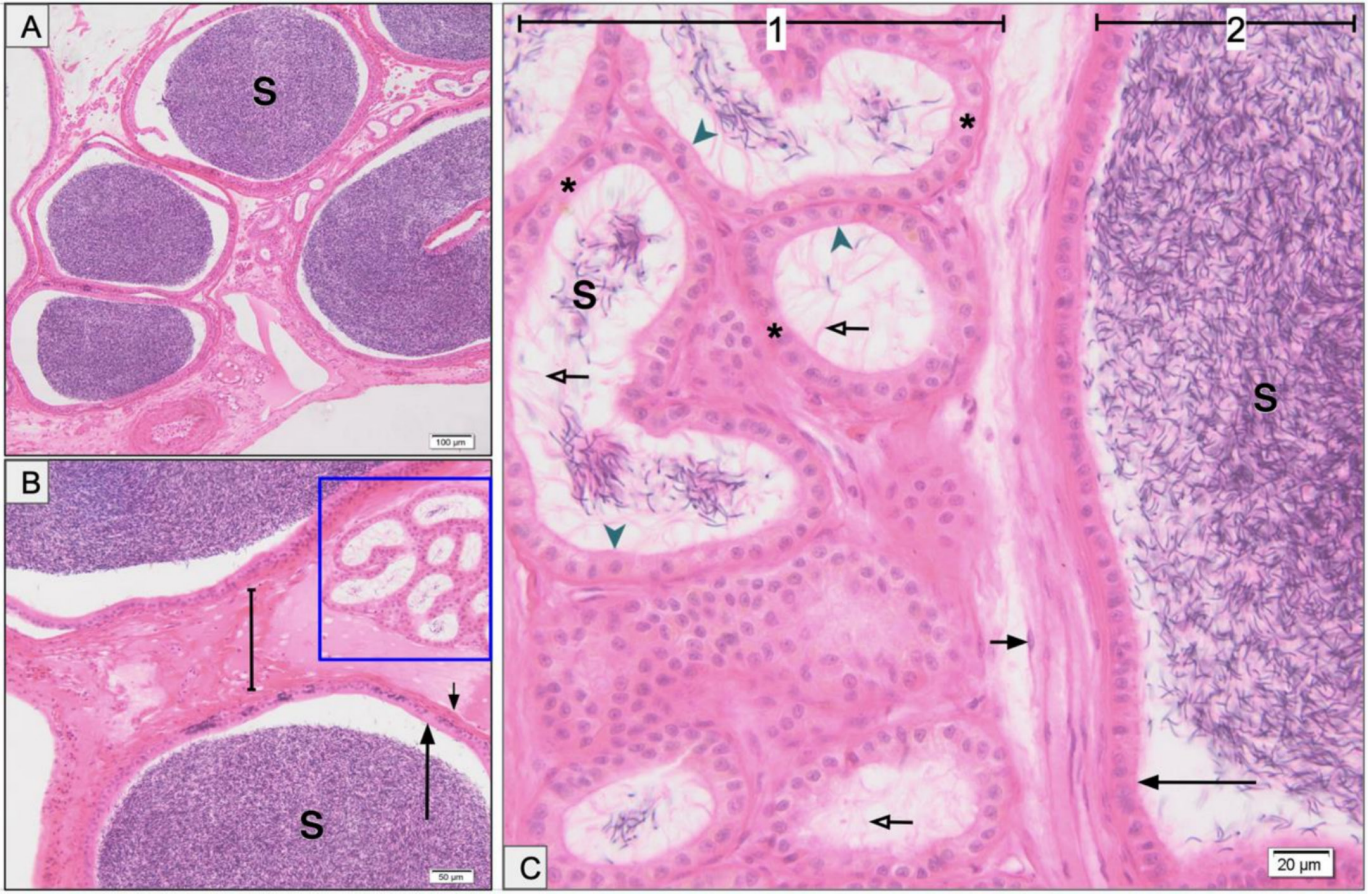
Histological structure of epididymis and deferent duct in *K. scorpioides* (H&E staining). (A) Epididymal lumen with abundant spermatozoa (S). (B) Convoluted tubules (box), connective tissue (line), fibromuscular tissue (short arrow), and simple columnar epithelium (long arrow). (C) Simple cuboidal epithelium (asterisk), basal lamina (arrowhead), stereocilia (hollow arrow), fibromuscular tissue (short arrow), simple columnar epithelium (long arrow). (1) Epididymis; (2) deferent duct. Scale bars: (A) 100 μm; (B) 50 μm; (C) 20 μm.

Histological sections stained with haematoxylin and eosin revealed the detailed architecture of the epididymis and deferent ducts (Figure 9). The ducts displayed distinct epithelial profiles, including simple columnar, simple cuboidal, and pseudostratified epithelia bearing stereocilia, corroborating the regional variation previously identified through ultrastructural analysis. Luminal spermatozoa were consistently observed, indicating active reproductive function at the time of collection.

**FIGURE 10:**
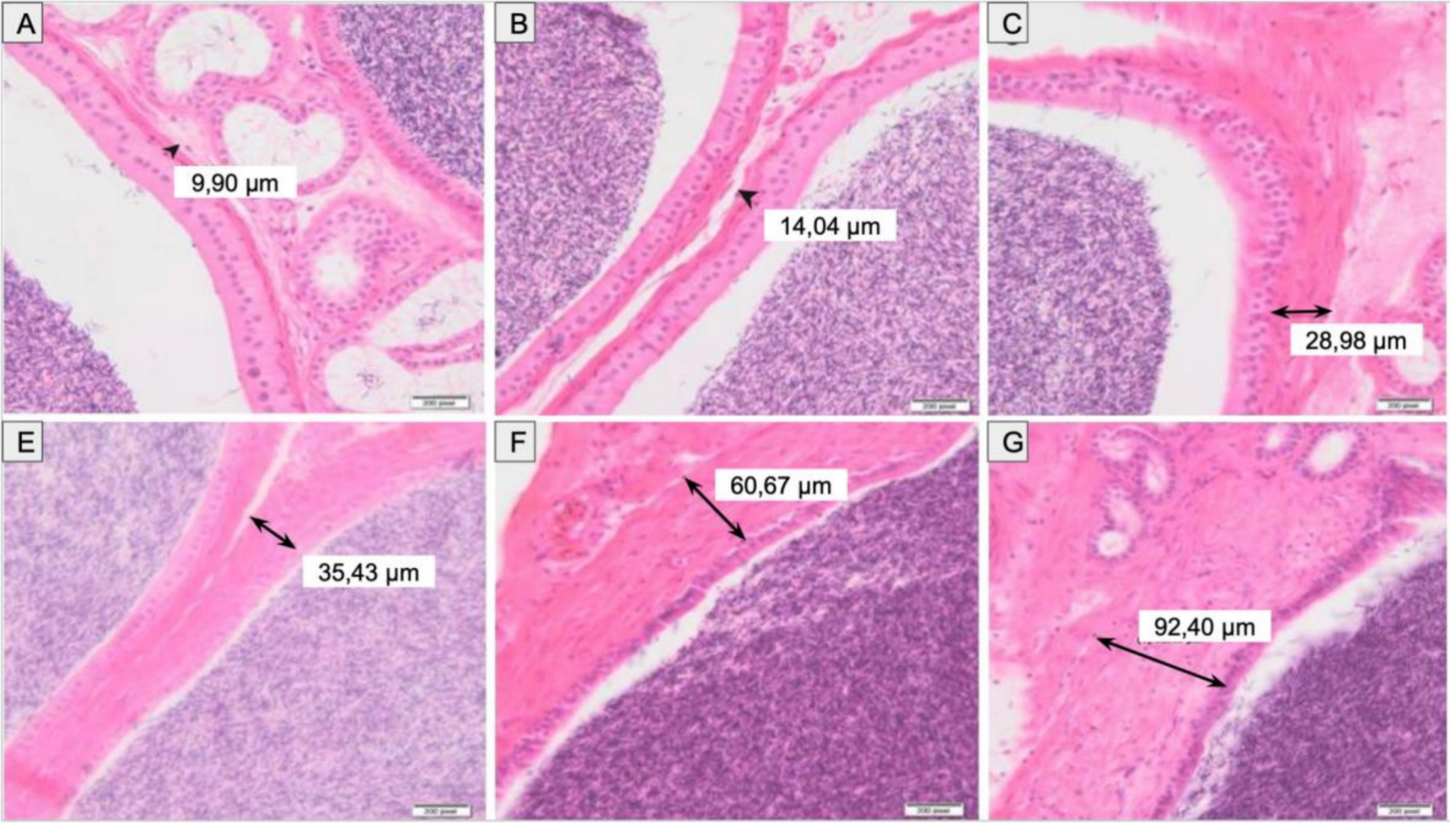
Comparative histology of the epididymis and deferent duct in *K. scorpioides* . Progressive increase in smooth muscle thickness from the epididymis to the deferent duct (H&E). Scale bars: (A–G) 200 μm.

The seminiferous tubules were densely packed with spermatozoa and enveloped by interstitial connective tissue. A progressive increase in smooth muscle thickness from the epididymis to the deferent ducts was noted, consistent with their respective roles in sperm propulsion and storage (Figure 10).

## 4 Discussion

Seasonally driven environmental fluctuations play a central role in shaping the reproductive strategies of *K. scorpioides* , a species that inhabits dynamic ecosystems such as Amazonian floodplains, Cerrado wetlands and Caatinga marginal habitats. The alternation between periods of high aquatic availability during the rainy season and severe hydric restriction during the dry season imposes selective pressures that have favoured the evolution of flexible reproductive physiology. Seasonal patterns of activity, including estivation‐like dormancy, have been described for this species (Pereira et al. 2014; Medeiros et al. 2024), and the present study provides anatomical and cellular evidence that reinforces the close link between environmental conditions and male reproductive function.

The presence of a fully active seminiferous epithelium, abundant germ cells at all stages of spermatogenesis, and lumina densely filled with spermatozoa observed in the present study indicates that January and February—months within the rainy season—correspond to a period of intense reproductive activation in *K. scorpioides* . This interpretation is further supported by the ultrastructural identification of spermatogonia, spermatocytes, round spermatids, and mature spermatozoa, confirming that testicular activity reaches its peak during months characterised by high rainfall, elevated temperatures, and increased primary productivity. These environmental conditions are known to synchronise courtship, mating, and nesting in many freshwater chelonians. In northern Brazil, the rainy season is characterised by increased temperature, rainfall, and humidity from January to July (Silva Da Silva, Stefany, et al. 2021), and a mating peak between January and June has been reported in the state of Pará in close association with these climatic factors (Costa et al. 2017). Moreover, our findings corroborate previous studies documenting increased testicular volume, elevated plasma testosterone concentrations, and marked morphological thickening of the genital ducts during this same period (Carvalho Viana, de Almei Anunciação, et al. 2014; Fernandes Araujo Chaves et al. 2020).

The epididymis exhibited marked epithelial heterogeneity, including simple cuboidal, simple columnar, and pseudostratified epithelia, with and without stereocilia. This mosaic‐like organisation suggests the existence of distinct regional specialisations analogous to the classical segmentation (caput–corpus–cauda) described in mammals, although such subdivisions are not macroscopically evident in reptiles. Similar epithelial complexity has been reported in crocodilians (Gribbins et al. 2010) and in soft‐shelled turtles such as *Pelodiscus sinensis* (Liu et al. 2016; Zhang et al. 2015), indicating a conserved structural organisation of the epididymal duct among reptilian lineages.

The presence of cilia or stereocilia is consistent with a role in facilitating sperm transport along the epididymal duct toward the ductus deferens (Fernandes Araujo Chaves et al. 2020). In the present study, this structural feature is congruent with the marked seasonal increase in testicular mass, suggesting elevated sperm production and a consequent demand for efficient epididymal transport mechanisms during the reproductive period.

In the deferent ducts, the observation of epithelial cells containing prominent nucleoli, abundant rough endoplasmic reticulum, and lipid inclusions indicates active synthesis and secretion during the reproductive peak. The progressive thickening of smooth muscle layers toward the distal duct is consistent with enhanced propulsive capacity, facilitating sperm release during mating. This structural pattern mirrors that reported in other turtles exhibiting seasonal reproduction, such as *Trachemys scripta* and *P. sinensis* , in which the ductus deferens acts as both a storage reservoir and a functional conduit during the breeding season.

The ultrastructural characteristics of spermatozoa observed here, well‐developed acrosomal vesicles, organised centriolar complexes, mitochondrial sheaths, and the canonical 9 + 2 axonemal arrangement, are consistent with patterns described in a variety of reptile species (Gribbins et al. 2010; Zhang et al. 2015). These conserved features underscore the evolutionary stability of the reptilian sperm architecture and reinforce its functional relevance to fertilisation success. Although this study did not evaluate female reproductive tracts, parallels with species known for long‐term sperm storage in the oviduct, such as *P. sinensis* (Liu et al. 2016), suggest that *K. scorpioides* may possess similar adaptations, enabling fertilisation after prolonged periods without mating. Further research targeting the female reproductive system will be essential to confirm this possibility.

From a conservation and management perspective, the biological patterns described here provide essential information for the design of reproductive monitoring programs and assisted reproductive technologies (ARTs). Identifying the rainy season as the period of maximal spermatogenic activity offers a practical guideline for optimising semen collection, cryopreservation, hormonal evaluation, and reproductive interventions in captive or threatened populations. The documentation of intact sperm architecture also establishes a foundation for future work on sperm quality, viability, motility, and responses to cryogenic protocols—critical steps for germplasm banking and artificial insemination programs, especially in populations affected by habitat fragmentation, pollution, and illegal harvesting (Berriozabal‐Islas et al. 2020; Rodrigues et al. 2017).

Finally, by integrating macroscopic, histological, and ultrastructural data, this study enhances current knowledge of the reproductive biology of *K. scorpioides* and highlights the species as a valuable model for understanding seasonal reproduction in freshwater turtles. The findings underscore the importance of linking ecological dynamics with reproductive physiology to better support species management, both in situ and ex situ. Future research incorporating hormonal profiling, functional sperm assays, and comparative seasonal sampling will refine our understanding of annual reproductive cycles and help strengthen conservation strategies for this and related chelonian species.

This study presents some limitations that should be considered when interpreting the results. First, all specimens were collected exclusively during the rainy season, which corresponds to the reproductive peak of *K. scorpioides* . Although this allowed a detailed characterisation of the active reproductive state, the absence of individuals sampled during the dry season prevents direct comparisons of seasonal regression, testicular quiescence, or changes in epididymal sperm storage throughout the annual cycle. Second, the sample size was modest and included one captive specimen, which may introduce biological variability related to environmental history, nutritional status, or stress exposure. Third, although TEM and SEM provided high‐resolution ultrastructural details, no functional assessments, such as sperm motility, viability, hormone levels, or quantitative morphometry, were performed. These parameters would further strengthen interpretations regarding reproductive performance and conservation applications. Despite these limitations, the combined macroscopic, histological, and ultrastructural analysis offers a valuable and novel dataset that contributes to the understanding of male reproductive biology in this species.

Taken together, these data provide the first integrated macroscopic, histological, and ultrastructural dataset for the male reproductive tract of the scorpion mud turtle collected during its reproductive season. The findings document active spermatogenesis, epididymal specialisation, and the presence of structurally intact spermatozoa across the reproductive tract, offering strong evidence for seasonal reproductive activation and short‐term sperm storage. The integration of LM, SEM, and TEM provides unprecedented detail for this species and fills a critical gap in chelonian reproductive anatomy. These contributions serve as a biological foundation for developing reproductive monitoring protocols, establishing fertility baselines, and informing assisted reproductive technologies for species management and conservation.

## 5 Conclusion

The findings presented here provide a detailed morphological characterisation of the male reproductive tract of *Kinosternon scorpioides* during the rainy season, demonstrating intensified spermatogenic activity, epididymal epithelial regionalization, and structurally preserved spermatozoa consistent with functional reproductive status. The data establish a reference framework for future comparative seasonal investigations and provide baseline information for reproductive assessment and conservation planning in freshwater chelonians.

## Conflicts of Interest

The authors declare no conflicts of interest.

## Data Availability

The data that support the findings of this study are available from the corresponding author upon reasonable request.
